# Environmental health centers for asbestos and their health impact surveys and activities

**DOI:** 10.1186/s40557-016-0154-8

**Published:** 2016-12-01

**Authors:** Dong-Mug Kang, Jong-Eun Kim, Yong-Jin Lee, Hyun-Hee Lee, Chang-yeol Lee, Seong-Jae Moon, Min-Sung Kang

**Affiliations:** 1 Environmental Health Center for Asbestos, Pusan National University Yangsan Hospital, Geumo-ro 20, Mulgeum-eup, Yangsan-si, Gyeongsangnam-do 50612 South Korea; 2 Preventive and Occupational Medicine, School of Medicine, Pusan National University, Yangsan, 50612 South Korea; 3 SoonChunhyang University Hospital Cheonan Asbestos Environmental Health Center, 31, Soonchunhyang 6-gil, Dongnam-gu, Cheonan-si, Chungcheongnam-do South Korea; 4 Department of Public Health, College of Health Science, Korea University, Seoul, South Korea; 5 Occupational and Environmental Medicine, Pusan National University Yangsan Hospital, 1st floor, 20, Geumo-ro, Mulgeum-eup, Yangsan-si, Gyeongsangnam-do 50612 South Korea

**Keywords:** Asbestos, Relief, Act, Environmental, Impact, Lung cancer, Mesothelioma, Awareness, Compensation

## Abstract

In 2009, Korea banned the import, transport, and use of asbestos, and the Asbestos Injury Relief Act (AIRA) was promulgated in 2011. Two environmental health centers for asbestos (EHCA), including Pusan National University Yangsan Hospital (PNUYH) and SoonChunHyang University Cheonan Hospital (SCHUCH), were adapted to find environmental asbestos-related diseases (ARDs) and to support the purposes of AIRA. EHCA conducted a health impact survey (HIS) on persons who resided or reside near asbestos factories or mines. A total of 13,433 persons have taken screening examinations in PNUYH EHCA, and 623 persons (4.6%) have had secondary examinations. Of the 21,014 persons who had screening examinations in SCHUCH EHCA, 2490 persons (11.8%) had secondary examinations. Some of those who tested positive for ARDs through HISs filed applications for the asbestos victims’ medical pocketbook (AVMP). Approximately 116 and 612 persons received AVMPs as a result of PNUYH and SCHUCH examinees, respectively. EHCAs have conducted HISs, public relations, and education for asbestos victims, ordinary citizens, and physicians. As HISs are based on voluntary participation, they does not monitor high-risk groups. Active surveillance focusing on high-risk groups has been blocked by the personal information protection act. Although important work has been performed in finding environmental asbestos victims and increasing public awareness on asbestos, it is necessary to improve the current system and registration.

## Background

Approximately 2–2.4 million tons of asbestos, including mined and imported raw asbestos material, have been used in Korea [1, 2]. In 2009, Korea banned the import, transport, and use of asbestos. Awareness of asbestos-related diseases (ARDs) including mesothelioma, lung cancer, and asbestosis as occupational and environmental diseases has a long history worldwide [3, 4]. After the first recognized case of occupational cancer to be caused by asbestos exposure occurred in an asbestos textile factory in 1993 [5], reports of occupational ARDs among construction and shipbuilding industries increased in Korea [6, 7]. Reports of other diseases, such as gastric cancer and laryngeal cancer, as ARDs have also been increasing in Korea [8, 9].

ARDs are recognized worldwide as environmental diseases. Because many Koreans are suffering from ARDs from environmental exposure, caused by inhaling asbestos released from asbestos factories and mines, the Asbestos Injury Relief Act (AIRA) was promulgated in 2011 and activated in 2012 [10]. The purpose of this act is to redress health damage caused by asbestos and to pay compensation to those affected [11]. Environmental Health Centers for Asbestos (EHCA), pursuant to Article 26 of the Environmental Health Act, were created to detect environmental ARDs and to support AIRA purposes. To this end, two EHCAs, Pusan National University Yangsan Hospital (PNUYH) and SoonChunHyang University Cheonan Hospital (SCHUCH), have been modified thus far. This paper details the activities and achievements of these two EHCAs.

## Health impact survey in suspected environmental asbestos exposure areas

There are two EHCAs in Korea. These organizations are designated pursuant to Article 26 of the Environmental Health Act. The EHCA conducted surveys concerning asbestos-related impacts on health (health impact survey: HIS) on persons who resided or reside near any of the following areas and who are likely or suspected to have sustained an injury caused by asbestos: 1) An area in which an asbestos mine has been operated; 2) An area in which asbestos or products containing asbestos have been manufactured or used in a large quantity; 3) Any other area prescribed in the ordinance of the Ministry of Environment’s AIRA [11]. These surveys were subsidized by AIRA’s asbestos relief fund (ARF). The subjects of the survey were residents who had lived within 2 km of the abovementioned areas for over 5 years. Table 1 shows a summary of the areas surveyed by the two EHCAs.

**Table 1:** Examinations area of EHCA

Year	PNUYH	SCHUCH
	Exposure	Exposure
2011	Asbestos factory (3)	Asbestos factory (2)
		Asbestos Mine (6)
2012	Asbestos Mine (1)	Asbestos Mine (17)
	Asbestos factory (4)	Asbestos loading dock (1)
		Quarry (1)
2013	Asbestos Mine (8)	Asbestos loading dock (1)
	Asbestos factory (4)	Asbestos Mine (15)
2014	Asbestos Mine (2)	Asbestos Mine (6)
	Asbestos factory (5)	Asbestos factory (3)
	Asbestos cement slate concentrated area (4)	Asbestos cement slate concentrated area (2)
		Asbestos loading dock (1)
2015	Asbestos factory (5)	Asbestos factory (8)
	Repair shipyard (2)	Asbestos Mine (2)
	Asbestos cement slate concentrated area (5)	Asbestos cement slate concentrated area (19)
2016	Asbestos Mine (1)	Asbestos Mine (5)
	Asbestos factory (11)	Asbestos factory (7)
	Asbestos cement slate concentrated area (5)	Asbestos loading dock (1)
	Repair shipyard (4)	

The HIS has two steps, a primary screening examination and a secondary close examination. Screening examinations include a physical examination by an occupational and environmental physician, a questionnaire, and a chest X-ray. In primary screenings, at least one of the following must be found before a doctor can perform a secondary examination: asbestosis and/or pleural plaques, which can be detected using a chest X-ray; abnormalities in the results of a pulmonary function test (PFT); or inspiratory crackles in the inferior lobe, which can be detected through auscultation. In the cases of abnormal findings from screening examinations, secondary close examinations are conducted. Close examinations include a chest CT scan and a PFT. The results of the examinations are reported to the Ministry of Environment (MoE). Individuals suspected of having ARDs are notified to file for recognition to receive remedial benefits recognized by the Korea Environment Corporation (KECO).

As AIRA was activated in 2012, until 2011 HISs were funded by the MoE and were conducted for research purposes. In addition to AIRA and MoE funding, since 2009 Busan metropolitan city government has funded HISs for Busan citizens. Table 1 shows the results of HISs. A total of 13,433 persons have taken screening examinations in PNUYH EHCA, and 623 persons (4.6%) have had secondary examinations. Among the 21,014 persons who had screening examinations in SCHUCH EHCA 2490 persons (11.8%) had secondary examinations.

## Recognition as asbestos victims as a result of HISs conducted by EHCAs

Some of those who were found to have ARDs through HISs filed applications with the district government, and the statistics for these are shown in Table 2. When KECO decides to recognize an applicant as an asbestos victim, an asbestos victims’ medical pocketbook (AVMP) is issued, and care, including remedial benefits, is provided. Of the PNUYH and SCHUCH examinees, approximately 116 and 612 persons received AVMPs, respectively. As there is no data on how many of the examinees applied for an AVMP, the rates of application and recognition cannot be calculated. Although the major purpose of AIRA is to provide relief for environmental victims, the majority of AVMP holders are former asbestos miners and individuals who worked high-risk jobs in the construction industry (data not shown).

**Table 2:** Results of health impact surveys by the environmental health centers for asbestos by year

Year	PNUYH				SCHUCH		Funding source
	Ministry of Environment		Busan Metropolitan City		Ministry of Environment		
	Screening Examination	Secondary Examination (%)	Screening Examination	Secondary Examination (%)	Screening Examination	Secondary Examination (%)	
2009	240	45 (18.8)	364	Not available	4497	886 (19.7)	Research
2010	520	6 (1.2)	276	21 (7.6)	4684	339 (7.2)	Research
2011	71	30 (42.3)	319	33 (10.3)	153	8 (5.2)	Research
2012	1208	57 (4.7)	553	53 (9.6)	1505	168 (11.2)	AIRA
2013	1250	41 (3.3)	506	53 (10.5)	2396	211 (8.8)	AIRA
2014	1339	140 (10.5)	620	86 (13.9)	2749	330 (12.0)	AIRA
2015	2551	93 (3.6)	581	67 (11.5)	2555	495 (19.4)	AIRA
2016 (Aug)	2571	5 (0.2)	464	12 (2.6)	2475	53 (2.1)	AIRA
Total	9750	417 (4.3)	3683	206 (5.6)	21,014	2490 (11.8)	

*AIRA* Asbestos Injury Relief Act

## Regular health check-ups for asbestos health care pocket book holders

People who have some signs of ARDs, such as pleural plaque, or who are suspected of having asbestosis but do not exhibit sufficient signs for a distinct diagnosis of asbestosis, can apply for an asbestos health care pocketbook (AHCP). AHCPs are issued to persons who are highly likely to have an ARD. People in receipt of AHCPs have periodic health examinations. Pocketbook holders can have periodic health check-ups within 2 year cycles; these involve the same steps and procedures as the screening and secondary examinations described above. The cycle can be shortened if the individual experiences aggravated symptoms. Also, they can receive influenza and pneumococcus vaccinations. Table 3 and 4 show the issue status AHCPs and asbestos health care pocketbooks by EHCAs (Table 3, Table 4).

**Table 3:** Asbestos victims’ medical pocketbooks provided as a result of HISs by EHCAs

Year	PNUYH		SCHUCH
	Ministry of environment	Busan Metropolitan	Ministry of environment
2011	0	1 (LC:1)	110 (MM:2, LC:5, As:103)
2012	0	2 (As:2)	112 (LC:8, As:104)
2013	14 (As:14)	13 (LC:1, As:12)	176 (LC:14, As:162)
2014	10 (As:10)	6 (As:6)	116 (MM:2, LC:7, As:106:, PT:1)
2015	23 (LC:1, As:22)	11 (As:11)	98 (LC:13, As:85)
2016 (Aug)	20 (As:20)	16 (As:16)	Not available
Total	67 (LC:1, As:66)	49 (LC:2, As:47)	612 (MM:4, LC:47, As:560, PT:1)

*As* Asbestosis, *LC* Lung cancer, *MM* Malignant mesothelioma, *PT* Pleural thickening

**Table 4:** Asbestos health care pocketbooks issued by EHCAs

Year	PNUYH		SCHUCH
	Ministry of Environment	Busan Metropolitan City	Ministry of Environment
2011	0	0	44
2012	0	1	90
2013	3	0	30
2014	4	1	60
2015	3	2	31
2016	0	1	32
Total	10	5	287

## Public relations and education

EHCAs offer social and information-sharing opportunities, eco tours of a national park, and a singing and laughing program to relax the body and mind. Asbestos awareness lectures by medical experts have been given to AVMP and AHCP holders and their relatives. Also, environmental health classes on asbestos have been held for residents around former (or present) asbestos factories and mines as well as shipyards. Presentations on HISs have been conducted for citizens in order to encourage participation in the screenings. Also, lectures on the legislation and systems that are in place for environmental asbestos victims have been given to physicians, including pulmonologists. Additionally, leaflets, booklets, and periodicals have been produced and distributed to pocketbook holders and citizens.

## Discussion

Major sources of environmental asbestos exposure are asbestos mines, asbestos textile factories, and the shipbuilding industry. The majority of asbestos mines were concentrated in Chungnam, and most asbestos textile factories were concentrated in Busan. Also, shipbuilding and repair industries are mainly located in Ulsan, Busan, and Gyeongnam [12]. These environmental exposure sources are major target areas for EHCAs’ HISs. PNUYH EHCA is in charge of the southern and eastern halves of Korea, whereas SCHUCH manages the northern and western halves of Korea. Hence, PNUYH concentrates on asbestos textile factories and shipbuilding companies, while SCHUCH focuses on asbestos mines and factories around the area of the capital. For SCHUCH, the main focus is Gwangcheon mine and Hongseong mine. PNUYH focuses on Jeil Chemical, which is an asbestos textile factory with many occupational victims located in residential areas in Busan [13]. As the main environmental asbestos sources for the patients of the two EHCAs differ, this means that their HIS results differ. Because of the long latency of ARDs it is difficult for people to realize that their diseases are consequences of previous asbestos exposure. As asbestos mines were located in rural areas with fixed residents, gathering and finding victims of mines could be relatively easier than in urban areas. As Korea experienced rapid economic growth, the urban population changed residential areas more frequently than residents of rural areas. This might explain the large differences between participants of HISs in the two EHCAs.

Based on the fact that the major ARDs worldwide are mesothelioma, lung cancer, and asbestosis [10], the afflictions of AVMP holders differ. Whereas asbestosis is predominant in AVMP holders, lung cancer is very rare. The rarity of lung cancer as an ARD might be explained by the strict criteria in place for recognizing lung cancer as an environmental as well as an occupational ARD [14]. On the basis of the current AIRA, an asbestosis or a pleural abnormality is essential for lung cancer to be recognized [11]. Although quantitative and qualitative exposure assessment and estimation should be a major means of recognizing environmental ARDs, it is a minimum requirement for relief. Exposure information is not a criterion for deciding whether lung cancer or asbestosis is related to environmental exposure. Hence, quantification of asbestos exposure without additional radiological findings only requires a single condition for lung cancer to be recognized.

As the number of asbestos victims recognized through HISs is small, the efficiency of HISs appears low. The HIS is based on voluntary participation. Considering persons with severe ARDs may have died or were unable to travel to the examination location, this voluntary HIS has limitations. There are high-risk groups or a vulnerable population who have experienced high exposure. Asbestos exposure during young age may have a higher potential to cause ARDs in the future [15]. The implementation of active surveillance that focuses on high-risk groups has been blocked by the personal information protection act. This barrier needs to be removed by clarifying the necessity for providing relief for asbestos victims through AIRA.

## Conclusion

EHCAs have conducted HISs, public relations, and provided education for asbestos victims, ordinary citizens, and physicians. However, the current survey system is based on voluntary participation, which has obvious disadvantages. An approach that utilizes a database of official residential information would be a better method of identifying high-risk groups than the present system. However, such a method would require a governmental system that makes use of residential identification records, a practice that is prohibited by the Personal Information Protection Act. It is necessary to improve the current system and registration process and to create and utilize an asbestos-victims database to provide proactive services for individuals who have suffered high levels of exposure to asbestos. Although EHCAs have played pivotal roles in finding environmental asbestos victims and in increasing public awareness of asbestos.

## Abbreviations

AHCP: Asbestos health care pocketbook
AIRA: Asbestos Injury Relief Act
ARD: Asbestos-related disease
ARF: Asbestos relief fund
AVMP: Asbestos victims’ medical pocketbook
ECHA: Environmental health centers for asbestos
HIS: Health impact survey
KECO: Korea Environment Corporation
MoE: Ministry of Environment
PFT: Pulmonary function test
PNUYH: Pusan National University Yangsan Hospital
SCHUCH: SoonChunHyang University Cheonan Hospital
